# Exercise-Induced Bronchoconstriction and Exercise-Induced Respiratory Symptoms in Nurses

**DOI:** 10.1155/2011/267542

**Published:** 2011-05-11

**Authors:** Jordan Minov, Jovanka Karadzinska-Bislimovska, Kristin Vasilevska, Snezana Risteska-Kuc, Saso Stoleski, Dragan Mijakoski

**Affiliations:** ^1^Department of Cardiorespiratory Functional Diagnostics, Institute for Occupational Health of R. Macedonia—WHO Collaborating Center and GA2LEN Collaborating Center, II Makedonska Brigada 43, 1000 Skopje, Macedonia; ^2^Institute for Epidemiology and Biostatistics, 1000 Skopje, Macedonia

## Abstract

In order to assess prevalence and characteristics of exercise-induced respiratory symptoms (EIRSs) and exercise-induced bronchoconstriction (EIB) in health care workers, we performed a cross-sectional study including 48 female nurses from primary care settings and an equal number of female office workers studied as a control group. The evaluation of examined groups included completion of a questionnaire, skin prick tests to common inhalant allergens, spirometry, and exercise and histamine challenge. We found a similar prevalence of EIRSs and EIB in both groups. EIB was closely related to asthma, atopy, family history of asthma, and positive histamine challenge in either group, while the association between EIB and daily smoking in nurses was of borderline statistical significance. Bronchial reaction to exercise was significantly higher in nurses than in controls with EIB. With the exception of exercise induced wheezing, EIRSs were weakly associated with EIB in both groups with a large proportion of false positive results.

## 1. Introduction

Exercise-induced bronchoconstriction (EIB), also referred to as exercise-induced asthma (EIA), is a manifestation of bronchial hyperresponsiveness (BHR) that occurs in the majority of patients with current symptomatic asthma, especially in the patients with moderately to severely increased responsiveness [1–3]. The current thinking about mechanisms by which EIB develops emphasizes the loss of heat and/or water from the airways during exercise that leads to release of proinflammatory mediators [4]. Airborne particles and pollutants, as well as airborne allergens, are considered as stimulants that contribute to EIB [5]. A fish oil supplementation may have a protective effect on EIB, which is probably attributed to its anti-inflammatory properties [6]. 

Results from several studies indicated that BHR prevalence is higher in females than in males [7, 8]. The mechanisms responsible for a higher susceptibility of the airways in females to nonspecific stimuli include lower airway calibre, lower body weight, greater cholinergic irritability, and hormonal factors [9]. 

On the other side, data from the studies carried out in the last two decades suggest an increased risk for asthma among health care workers, yet only a few specific determinants have been elucidated [10–13]. As Delclos et al. [14] have suggested, the contribution of occupational exposures to respiratory impairment and asthma in health care professionals is not trivial, meriting both implementation of appropriate controls and further studies. 

To our knowledge, so far, there is no study assessing exercise-induced respiratory symptoms (EIRSs) and EIB in health care professionals. In the present study, we assessed effects of occupational exposure on EIRSs and EIB among health care workers by comparison of their prevalence and characteristics between females working as nurses in primary care settings and female office workers. 

## 2. Materials and Methods

### 2.1. Study Design and Setting

A cross-sectional survey was carried out in a university research laboratory, that is, Department of Cardiorespiratory Functional Diagnostics at the Institute for Occupational Health of R. Macedonia, Skopje—WHO Collaborating Center for Occupational Health and GA^2^LEN Collaborating Center.

### 2.2. Subjects

We examined 48 females aged 24 to 51 years (mean age 37.8 ± 7.4) working as nurses in primary care settings with duration of employment 5 to 25 years (mean duration 14.7 ± 5.7). 

The work shifts of the nurses lasted 8 hours per day, and their working tasks included completion of the medical documentation, assistance in medical interventions, administration of parenteral and aerosolized medications, and medical instruments cleaning. The workplace exposure included several types of cleaning products, disinfectants, adhesives, solvents, latex, and medications, some of which are in spray form. According to the classification of occupational muscular work, their work was classified as a light muscular work [15]. During the work shift, they use protective clothing, masks, and powdered latex gloves. 

In addition, an equal number of female office workers matched to nurses as a group by age and smoking status were studied as a control group. According to the classification of occupational muscular work, their work was classified as a sedentary work. 

In either group, there were no subjects in whom exercise challenge or histamine challenge were contraindicated [16, 17], nor there were subjects with the upper respiratory viral infection within three weeks before the challenge test was performed. None of the subjects took asthma medications or antihistamines at least one month before the challenge tests and skin-prick tests. Daily smokers were asked to restrain from smoking at least 3 hours before testing.

### 2.3. Questionnaire

The questionnaire was designed using the proposed model of the National Jewish Medical and Research Center, Denver, USA [18].

Subjects were considered having exercise-induced respiratory symptoms (EIRSs) if one or more symptoms were reported: coughing during or after exercise, wheezing during or after exercise, inability to get deep breath after exercise, noisy breathing after exercise, and chest tightness after exercise.

Detailed smoking history, asthma diagnosed by physician, family history of asthma and allergic diseases (taking into account the first-degree relatives), accompanying disease, and medication use were also evaluated.

Classification of smoking status was done according to the World Health Organization (WHO) guidelines on definitions of smoking status [19].

Daily smoker was defined as a subject who smoked at the time of the survey at least once a day, except on days of religious fasting. In daily smokers, lifetime cigarette smoking and daily mean of cigarettes smoked were evaluated. Pack years smoked (one pack year denotes one year of smoking 20 cigarettes per day) were calculated according to the actual recommendations [20]. 

Ex-smoker was defined as a formerly daily smoker who no longer smokes.

Passive smoking or exposure to environmental tobacco smoke (ETS) was defined as the exposure of a person to tobacco-combustion products from smoking by others [21].

### 2.4. Skin-Prick Tests

Skin-prick tests (SPTs) to common inhalant allergens were performed in all subjects on the volar part of the forearm using commercial allergen extracts (Torlak, Serbia, and Montenegro) of birch (5000 PNU), grass mixed (5000 PNU), plantain (5000 PNU), fungi mixed (4000 PNU), *Dermatophagoides pteronyssinus* (3000 PNU), dog hair (4000 PNU), cat fur (4000 PNU), and feathers mixed (4000 PNU). All tests included positive (1 mg/mL histamine) and negative (0.9% saline) controls. Prick tests were considered positive if the mean wheal diameter 20 min after allergen application was at least 3 mm larger than the size of the negative control [22]. Atopy was defined as the presence of at least one positive SPT [23].

### 2.5. Spirometry

Spirometry, including measures of forced vital capacity (FVC), forced expiratory volume in one second (FEV_1_), FEV_1_ / FVC ratio, and maximal expiratory flow at 50%, 25%, and 25–75% of FVC (MEF_50_, MEF_25_, and MEF_25–75_, resp.), was performed in all subjects using spirometer Ganshorn SanoScope LF8 (Ganshorn Medizin Electronic GmbH, Germany) with recording the best result from three measurements the values of FEV_1_ of which were within 5% of each other. The results of spirometry were expressed as percentages of the predicted values according to the European Community for Coal and Steel (ECCS) norms [24].

### 2.6. Histamine Challenge

The histamine challenge test was performed according to the actual European Respiratory Society (ERS)/American Thoracic Society (ATS) recommendations [16, 17]. Concentrations of 0.5, 1, 2, 4, and 8 mg/mL histamine (Torlak, Beograd) were prepared by dilution with buffered saline. The doses of aerosol generated by Pari LC nebulizer with output rate 0.17 mL/min were inhaled by mouthpiece. Subjects inhaled increasing concentrations of histamine using a tidal breathing method until FEV_1_ fell by more than 20% of its base value (provocative concentration 20—PC20) or the highest concentration was reached. 

According to the ATS recommendations, bronchial hyperresponsiveness (BHR) was categorized as moderate to severe BHR (PC20 < 1.0 mg/mL), mild BHR (PC20 = 1.0–4.0 mg/mL), and borderline BHR (PC20 > 4.0 mg/mL) [17]. The test was considered positive if PC20 was equal or less than 4 mg/mL [16, 17].

### 2.7. Exercise Challenge Tests

The constant submaximal exercise challenge test (ECT) was performed in all subjects using cycle ergometer Hellige-dynavit Meditronic 40 (Hellige GmbH, Germany). ECT was conducted in an air-conditioned room with ambient temperature of 20–25°C and relative air humidity of 50% or less. According to the actual recommendations, subjects exercised 8–10 min achieving 90% of predicted maximal heart rate (HR_max_ = 220 − age) in the last 4 min of exercise [16, 17]. Heart rate was monitored continuously throughout the exercise and for 5 minutes after its completion from a three-lead electrocardiographic configuration. The measurements of FEV_1_ were performed before and 1, 3, 5, 7, 10, and 15 min after the exercise with inhaled bronchodilator (200 mcg salbutamol) application upon completion of the protocol.

The response to exercise was expressed as fall index FEV_1_ (100 × [pre-exercise FEV_1_ − lowest postexercise FEV_1_]/pre-exercise FEV_1_). EIB was defined as fall index FEV_1_≥ 10% [16]. 

### 2.8. Statistical Analysis

Continuous variables were expressed as mean values with standard deviation (SD) whereas the nominal variables as numbers and percentages. Analyses of the data involved testing the differences in prevalence, comparison of the means, and testing the association between EIRSs and EIB and studied variables. Chi-square test was used for testing difference in the prevalence. Comparison of spirometric measurements and fall index FEV_1_ values was performed by independent samples *t*-test. Chi-square test (or Fisher's exact test where appropriate) was used for testing association between EIRSs and EIB and studied variables. A *P* value less than  .05 was considered as statistically significant. Statistical analysis was performed using the Statistical Package for the Social Sciences (SPSS) version 11.0 for Windows.

## 3. Results

Demographic characteristics of the study subjects were similar in both examined groups (Table 1).

Prevalence of EIRSs, total and individual, was similar in both examined groups. Inability to get deep breath after exercise and cough during or after exercise was the most frequent EIRSs in either group (Table 2).

EIRSs were nonsignificantly associated with age and smoking in both examined groups. The association between EIRSs in nurses and duration of employment was also nonsignificant.

Prevalence of subjects with positive SPT to common inhalant allergens was similar in both nurses and controls (33.3% versus 37.5%, *P* = .670; Chi-square test). Mite sensitization was the most important individual common allergen with no statistical difference between sensitized subjects in both groups (22.9% versus 25.0%, *P* = .811; Chi-square test). 

Spirometric parameters were lower in nurses, but statistical significance was not found for any parameter (Table 3). Spirometric parameters were nonsignificantly lower in the subjects with asthma diagnosed by physician as compared to nonasthmatics in both nurses and controls. 

Prevalence of overall subjects with BHR was nonsignificantly higher in nurses (12.5% versus 8.3%; *P* = .740), whereas the prevalence of subjects with moderate to severe and mild BHR, that is, prevalence of subjects with positive histamine challenge, was similar in both examined groups (Table 4). 

We found similar prevalence of EIB in both nurses and controls (8.3% versus 6.3%, *P* = 1.000; Fisher's exact test). The EIB severity, expressed as fall index FEV_1_, was significantly higher in nurses (28.1% versus 22.7%, *P* = .033; independent-samples *t*-test). Characteristics of the ECT performed in study subjects are shown Table 5.

EIB in both examined groups was significantly related to asthma diagnosed by physician, positive family history for asthma and allergies, and positive histamine challenge, whereas association with other variables was nonsignificant. Association between EIB and daily smoking in nurses was of borderline statistical significance (*P* = .062; Fisher's exact test), while association between EIB and pack years smoked (less or more than 12) was nonsignificant (*P* = .097; Fisher's exact test). These associations in controls were statistically nonsignificant. 

Association between EIB and exercise-induced respiratory symptoms, with exception of exercise-induced wheezing in both nurses (*P* = .037; Fisher's exact test) and controls (*P* = .034; Fisher's exact test), was statistically nonsignificant. The frequency of false positive results was high in both nurses (84.3%) and controls (88.2%).

## 4. Discussion

According to the recent data, occupational exposures in health care professionals increase the risk of work-related asthma. Medical instruments cleaning, general cleaning, use of solvents/adhesives in patient care, use of powdered latex gloves, and aerosolized medication administration were identified as occupational risk factors associated with the development of asthma in nurses [10, 14, 25]. 

On the other hand, EIB is a common condition close related to asthma that is often unrecognized and uncontrolled leading affected subjects to avoid general and occupational physical activities and sports. We performed the present study on EIB among nurses in primary care settings as a continuum of our investigations on the effects of specific occupational exposures on the EIB occurrence and characteristics [26–28]. According to the results of several studies [11–13], the lowest risk of respiratory impairment and asthma was found in administrative workers, so this “unexposed” occupation was used as a control group. 

In the present study, both examined groups included subjects with similar demographic characteristics. In either group, there was a large proportion of daily and passive smokers similar to its prevalence among females in R. Macedonia documented in our previous studies [29, 30]. The prevalence of ex-smokers in both groups was low, suggesting insufficient smoking cessation activities. The situation in the developed countries seems to be somewhat different. In the study conducted in 12 European countries as well as Australia and the USA, Janson et al. [31] reported that both active and passive smoking rates have declined since the early 1990s but indicated lower quitting rates and higher risk of passive smoking among people with fewer qualifications and less skilled occupation groups. 

We found high prevalence of EIRSs in both examined groups that is similar to the findings of several studies which investigated EIB in different subpopulations of both sexes [32, 33] as well as to the findings of our studies among workers with different occupational exposures [26–28]. The prevalence of atopy and the pattern of allergic sensitization to common aeroallergens in both examined groups was comparable to that we had previously observed among adults in R. Macedonia [34, 35]. All spirometric parameters were lower in nurses, but statistical significance was not achieved for any of them. The prevalence of BHR was nonsignificantly higher in nurses than in office workers that is similar to the findings obtained in our previous studies on BHR prevalence among workers with specific occupational exposures (herbal tea processors, cooks, and cleaners) and office workers as a control group [36, 37]. 

Several studies indicated that the occurrence of EIB depends on degree of bronchial hyperresponsiveness (alias underlying chronic inflammation), exercise intensity, and ambient conditions [38, 39]. There are many studies about EIB occurrence in selected groups of general population (children, school children, adolescents, and recruits) as well as in recreative and elite athletes. On the contrary, there is a limited number of studies on EIB associated with specific workplace exposures. The EIB prevalence in elite athletes varies from 12% of basketball players to 55% of cross-country skiers [40, 41]. In the present study, we found similar EIB prevalence in both nurses and controls (8.3% and 6.3%, resp.). According to the results of our previous studies, the EIB prevalence among workers with specific occupational exposures ranged from 6.4% in herbal tea processors, 6.9% in bakers, 7.1% in agricultural workers, 8.9% in textile workers, to 9.3% in agricultural workers. Bronchial reaction to exercise in the subjects with EIB was significantly higher in nurses than in controls. Significantly higher bronchial reaction to exercise in comparison to office workers we also found in ECT-positive female cleaners, whereas the difference in mean fall index FEV_1_ did not differ significantly between workers exposed to organic dusts and office workers with EIB [26–28]. This difference may be due to the presence of the study subjects of both sexes among workers exposed to organic dusts as well as to the different occupational exposures (i.e., dominant exposure to chemical compounds in cleaners and nurses).

We found significant association between EIB and asthma, family history of asthma, and atopy in both examined groups. Contribution of genetic factor in the EIB development is confirmed in a number of studies [39, 42, 43]. We also found a significant association between positive ECT and positive histamine challenge in both examined groups. Data from the studies which compared results of two bronchial challenge types are somewhat inconsistent. Some authors reported significant association between the results of exercise and histamine challenge [26–28, 44]. On the contrary, other authors reported a weak association, which was explained by different pathomechanisms of BHR to histamine and EIB [45]. Correlation between EIB and daily smoking was nonsignificant in controls, whereas in nurses, it was of borderline statistical significance. A similar finding, suggesting possible interaction of tobacco smoke and occupational exposure in EIB development, was obtained in our previous study on EIB in female cleaners [28]. 

In the present study, there was no positive association in both examined groups between EIB and overall and individual EIRSs with exception of exercise-induced wheezing. This finding, confirmed in a number of studies, was not unexpected, as the EIRSs may be triggered by many conditions and diseases other than BHR (e.g., physical unfitness, medical side effect of angiotensin-converting enzyme inhibitors and beta-blockers, anxiety, vocal cord dysfunction, arterial hypertension, gastroesophageal reflux, etc.) [26–28, 46–48].

The present study has some limitations. First, relatively smaller number of the subjects in the study groups could have certain implications on the data obtained and its interpretation. Second, we did not perform SPT to workplace allergens (e.g., latex), so we could not document relationship between sensitization to workplace allergens and EIB. Third, environmental measurements were not performed, so we could not document the effect of the type and the level of exposure on EIB. The strength of the study is the extensive examination of lung function in the study subjects with the possibility for comparison of the results of different tests. 

## 5. Conclusions

In conclusion, in a cross-sectional study including nurses and office workers, we found a similar prevalence of EIRSs and EIB in both examined groups. EIB was strongly related to asthma in both nurses and controls. In addition, EIB was closely related to atopy, family history of asthma, and positive histamine challenge in either group as well as to daily smoking in nurses. Bronchial reaction to exercise in ECT-positive nurses was significantly higher than in ECT-positive controls. EIRSs were weakly associated with EIB in both examined groups, with a large proportion of false positive results. Our study confirms the need of regular medical examinations in order to identify affected workers and to implement adequate preventive measures. 

## Figures and Tables

**Table 1:** Demographics of the study subjects.

Variable	Nurses	Office workers
	(*n* = 48)	(*n* = 48)
Age (years)	37.8 ± 7.4	39.1 ± 9.2
BMI (kg/m^2^)	25.4 ± 3.9	26.7 ± 4.3
Duration of employment (years)	14.7 ± 5.7	15.4 ± 7.8
Asthma diagnosed by physician	3 (6.3%)	2 (4.2%)
Family history of asthma	4 (8.3%)	4 (8.3%)
Family history of allergies	6 (12.5%)	8 (16.6%)
Daily smokers	14 (29.2%)	15 (31.2%)
Smoking experience (years)	17.7 ± 5.8	19.4 ± 7.9
Cigarettes per day	14.4 ± 6.9	16.8 ± 8.3
Pack years smoked	12.5 ± 3.1	13.4 ± 3.8
Daily smokers with less than 12 pack years smoked	6 (12.5%)	7 (14.6%)
Ex-smokers	5 (10.4%)	4 (8.3%)
Passive smokers	8 (16.6%)	10 (20.8%)

Numerical data are expressed as mean value with standard deviation and frequencies as number and percentage of study subjects with certain variable.

BMI: body mass index; kg: kilogram; m: meter.

**Table 2:** Prevalence of EIRSs in nurses and controls.

EIRSs	Nurses	Office workers	*P* value*
	(*n* = 48)	(*n* = 48)	
Any exercise-induced respiratory symptom	19 (39.6%)	17 (35.4%)	.673
Cough	11 (22.9%)	13 (27.1%)	.637
Inability to get deep breath	14 (29.1%)	11 (22.9%)	.585
Wheezing	5 (10.4%)	4 (8.3%)	1.000
Chest tightness	9 (18.8%)	8 (16.7%)	.726
Noisy breathing	4 (8.3%)	6 (12.5%)	.740

Data are expressed as number and percentage of study subjects with certain variable.

EIRSs: exercise-induced respiratory symptoms.

*Tested by Chi-square test (or Fisher's exact test where appropriate).

**Table 3:** Spirometric parameters in the study subjects.

Spirometric parameter	Nurses	Office workers	*P* value*
	(*n* = 48)	(*n* = 48)	
FVC (% pred)	88.9 ± 9.8	91.6 ± 10.4	.102
FEV_1_ (% pred)	84.3 ± 7.9	86.2 ± 9.6	.180
FEV_1_/FVC%	76.1 ± 4.9	78.4 ± 5.8	.126
MEF_50_ (% pred)	64.8 ± 12.7	68.1 ± 9.8	.083
MEF_25_ (% pred)	60.8 ± 10.1	64.9 ± 8.9	.069
MEF_25–75_ (% pred)	72.8 ± 14.7	75.1 ± 10.9	.094

Data are expressed as mean value with standard deviation.

FVC: forced vital capacity; FEV_1_: forced expiratory volume in one second; MEF_50_, MEF_25_, and MEF_25–75_: maximal expiratory flow at 50%, 25%, and 25–75% of FVC, respectively; % pred: % of predicted value.

*Compared by independent samples *t *test.

**Table 4:** Characteristics of the ECT in nurses and controls.

BHR	Nurses	Office workers	*P* value*
	(*n* = 48)	(*n* = 48)	
Moderate to severe BHR	2 (4.2%)	1 (2.1%)	1.000
Mild BHR	1 (2.1%)	1 (2.1%)	1.000
Borderline BHR	3 (6.3%)	2 (4.2%)	1.000

Data are expressed as mean value with standard deviation.

BHR: bronchial hyperresponsiveness.

*Compared by Fisher's exact test.

**Table 5:** Characteristics of the ECT in nurses and controls.

Variable	Nurses	Controls
	(*n* = 48)	(*n* = 48)
Exercise load (Watt)	102.1 ± 20.3	106.3 ± 16.1
Positive ECT	4 (8.3%)	3 (6.3%)
ΔFEV_1_ in the subjects with EIB (%)	28.1 ± 3.4	22.7 ± 1.5
Time of EIB occurrence (minutes after exercise)	6.1 ± 2.1	6.9 ± 2.7

Numerical data are expressed as mean value with standard deviation and frequencies as number and percentage of study subjects with certain variable.

ECT: exercise challenge test; ΔFEV_1_: a fall in FEV_1_ of pre-exercise value; FEV_1_: forced expiratory volume in 1 second; EIB: exercise-induced bronchoconstriction.
